# A Conserved Residue, Tyrosine (Y) 84, in H5N1 Influenza A Virus NS1 Regulates IFN Signaling Responses to Enhance Viral Infection

**DOI:** 10.3390/v9050107

**Published:** 2017-05-12

**Authors:** Ben X. Wang, Lianhu Wei, Lakshmi P. Kotra, Earl G. Brown, Eleanor N. Fish

**Affiliations:** 1 Toronto General Hospital Research Institute, University Health Network, 67 College Street, Toronto, ON M5G 2M1, Canada; ben.wang@mail.utoronto.ca (B.X.W.); william.wei@utoronto.ca (L.W.); lkotra@uhnresearch.ca (L.P.K.); 2 Department of Immunology, University of Toronto, 1 King’s College Circle, Toronto, ON M5S 1A8, Canada; 3 Center for Molecular Design and Preformulations, University Health Network, 101 College Street, Toronto, ON M5G 1L7, Canada; 4 Department of Pharmaceutical Sciences, Leslie Dan Faculty of Pharmacy, University of Toronto, 144 College Street, Toronto, ON M5S 3M2, Canada; 5 Department of Biochemistry, Microbiology and Immunology, Faculty of Medicine, University of Ottawa, 451 Smyth Road, Ottawa, ON K1H 8M5, Canada; ebrown@uottawa.ca

**Keywords:** influenza A viruses, non-structural protein 1, interferon-β, interferon signaling, interferon-stimulated genes

## Abstract

The non-structural protein, NS1, is a virulence factor encoded by influenza A viruses (IAVs). In this report, we provide evidence that the conserved residue, tyrosine (Y) 84, in a conserved putative SH2-binding domain in A/Duck/Hubei/2004/L-1 [H5N1] NS1 is critical for limiting an interferon (IFN) response to infection. A phenylalanine (F) substitution of this Y84 residue abolishes NS1-mediated downregulation of IFN-inducible STAT phosphorylation, and surface IFNAR1 expression. Recombinant IAV (rIAV) [H1N1] expressing A/Grey Heron/Hong Kong/837/2004 [H5N1] NS1-Y84F (rWSN-GH-NS1-Y84F) replicates to lower titers in human lung epithelial cells and is more susceptible to the antiviral effects of IFN-β treatment compared with rIAV expressing the intact H5N1 NS1 (rWSN-GH-NS1-wt). Cells infected with rWSN-GH-NS1-Y84F express higher levels of IFN stimulated genes (ISGs) associated with an antiviral response compared with cells infected with rWSN-GH-NS1-wt. In mice, intranasal infection with rWSN-GH-NS1-Y84F resulted in a delay in onset of weight loss, reduced lung pathology, lower lung viral titers and higher ISG expression, compared with mice infected with rWSN-GH-NS1-wt. IFN-β treatment of mice infected with rWSN-GH-NS1-Y84F reduced lung viral titers and increased lung ISG expression, but did not alter viral titers and ISG expression in mice infected with rWSN-GH-NS1-wt. Viewed altogether, these data suggest that the virulence associated with this conserved Y84 residue in NS1 is, in part, due to its role in regulating the host IFN response.

## 1. Introduction

H5N1 avian influenza A viruses (IAVs) that infect poultry and migratory birds pose a significant threat to global health and, since 2003, there have been 858 confirmed cases of H5N1 IAV infection in humans with a mortality rate of 53% [1]. While annual vaccines are effective in preventing seasonal IAV infections, they have limited use in the event of an outbreak of a newly emergent strain. Currently, the neuraminidase inhibitors oseltamivir (Tamiflu) and zanamivir (Relenza) are antivirals available to treat IAV infections. However, drug-resistant strains of IAVs, including pandemic H1N1 and H5N1, have been isolated [2,3,4].

Given the direct antiviral and immunomodulatory effects of interferons (IFNs)-α/β [5] and the importance of the innate immune response for limiting viral infection and spread [6], IFNs present as candidate broad-spectrum antivirals with the potential to act as a first-line treatment for existing and newly emergent IAV infections [7,8]. In a previous report, we provided evidence for the antiviral effects of IFN-α in limiting H5N1 and pandemic H1N1 2009 IAV replication in primary human lung cells [9]. Moreover, mice lacking a functional type I IFN receptor, IFNAR, exhibit significantly more weight loss and a more rapid time to death when infected with various IAVs including H5N1 and H1N1 subtypes, compared with mice with an intact IFN system [10,11]. Not surprisingly, IAVs have evolved mechanisms to evade and disrupt host IFN production, IFN signaling and IFN-inducible antiviral effector functions [9,12].

The non-structural protein 1 (NS1) is a virulence factor encoded by IAVs and is expressed in the nucleus and cytoplasm of host cells during the earliest stages of infection [13,14]. Functional as a dimer, NS1 is comprised of an N-terminal dsRNA-binding domain and a C-terminal protein-binding effector domain [15,16,17]. In the context of limiting an IFN response to infection, NS1 inhibits IFN-β production by preventing the activation of retinoic acid-inducible gene 1 (RIG-I) products [18,19]. In addition, NS1 can prevent the maturation of host mRNAs, including IFN-α/β mRNAs, by binding to and inhibiting cleavage and polyadenylation specific factor 4, 30 kDa subunit (CPSF4), and poly(A)-binding protein II (PABPII) [20,21]. Consequently, IAVs lacking NS1, or expressing truncated forms of NS1, induce higher levels of IFN-α/β mRNA expression and IFN production, and have been proposed as live-attenuated vaccines [22,23,24]. In addition to inhibiting the production of IFNs-α/β, in an earlier publication we provided indirect evidence that IAVs may also limit IFN signaling, mediated by NS1 disrupting IFN-inducible phosphorylation of signal transducer and activator of transcription (STAT) 1 and STAT2 [9].

The IAV NS1 N-terminal effector domain contains a Src homology (SH)3 and a putative SH2-binding motif, that are important for direct binding with p85β, the inhibitory subunit of phosphatidylinositol-3-kinase (PI3K) [25,26,27]. Binding of NS1 to the internal SH2 (i-SH2) domain of p85β leads to the activation of the PI3K-protein kinase B (AKT) pathway, to enhance viral replication. A tyrosine (Y) to phenylalanine (F) substitution at the strictly conserved residue 89 (Y89F) in the H1N1 NS1 putative SH2-binding domain prevented binding of NS1 to p85β, thus abrogating NS1-mediated AKT phosphorylation [25,26]. Additionally, this Y89F in the NS1 of IAV PR8 reduced virulence in infected mice [28].

SH2 domains are well-conserved motifs found in many intracellular signaling proteins, such as those responsible for initiating IFN-α/β signaling pathways [29,30] and may present as targets for NS1–host protein interactions that affect the host innate immune response to IAV infection. In this study, we used site-directed mutagenesis to alter the conserved Y84 residue within H5N1 NS1 in order to characterize its role in limiting the host IFN signaling response. In the context of IAV infection, we used reverse genetics to generate recombinant IAVs (rIAVs) [H1N1] encoding either a wildtype or mutant H5N1 NS1 and confirmed the importance of this putative SH2-binding domain for virus replication, providing evidence for its contribution to evasion of the host IFN response.

## 2. Materials and Methods

### 2.1. Cells and Reagents

Human cervical carcinoma HeLa cells, lung adenocarcinoma epithelial A549 cells, embryonic kidney HEK293T cells, Madin-Darby canine kidney (MDCK) cells, and mouse embryonic fibroblasts (MEFs) were purchased from ATCC (Manassas, VA, USA). STAT1^+/+^ and STAT1^−/−^ MEFs were provided by Dr. Leonidas C. Platanias (Robert H. Lurie Comprehensive Cancer Center, Chicago, IL, USA). All cells were maintained in Dulbecco’s modified Eagle’s medium (DMEM) supplemented with 10% fetal calf serum (FCS), 100 U/mL penicillin, and 100 μg/mL streptomycin (Invitrogen, Waltham, MA, USA) at 37 °C and 5% CO_2_.

Human IFN-β-1a (Avonex, specific activity 1.2 × 10^7^ U/mL), murine IFN-β1 (specific activity 3.6 × 10^7^ U/mL), and an anti-human IFNAR1 antibody (unconjugated, clone AA3) were provided by Darren P. Baker (BiogenIdec, Cambridge, MA, USA). An anti-human IFNAR2 antibody (unconjugated, clone MMHAR-2) was purchased from PBL Assay Science (Piscataway, NJ, USA). An anti-mouse IgG (Alexa Fluor 647, H+L) was purchased from Invitrogen as a secondary antibody. Antibodies specific for human phospho (p)-AKT (Ser473), AKT, p-STAT1 (Tyr701), STAT1, p-STAT2 (Tyr690), STAT2, and HA-Tag (6E2) were purchased from Cell Signaling Technology (Danvers, MA, USA). An antibody specific for human α-tubulin was purchased from Sigma-Aldrich (St. Louis, MO, USA) and horseradish peroxidase (HRP)-conjugated anti-rabbit IgG and anti-mouse IgG secondary antibodies were purchased from GE Healthcare Life Sciences (Marlborough, MA, USA). Antibodies specific for mouse CD11b (BV421, clone M1/70) and CD45 (BV605, clone 30-F11) were purchased from BioLegend (San Diego, CA, USA). Antibody specific for mouse Ly6G was purchased from eBioscience (San Diego, CA, USA). Respective isotype control antibodies were purchased from BioLegend and eBioscience.

### 2.2. Mice

Male C57BL/6 mice, aged 6–8 weeks, were purchased from Taconic (Hudson, NY, USA) and housed in a pathogen-free environment. All experiments were approved by the Animal Care Committee of the Toronto General Hospital Research Institute.

### 2.3. In Silico Modeling

The crystallized structure of A/Puerto Rico/8/1934 [H1N1] NS1 and p85β i-SH2 domain complex (RCSB Protein Data Bank: 3L4Q) [31] was used to construct a model of avian A/Vietnam/1203/2004 [H5N1] NS1 (RCSB Protein Data Bank: 3F5T) [15] and p85β i-SH2 domain complex using SYBYL-X (Certara, Princeton, NJ, USA). The NS1 subunit from 3L4Q was removed and replaced with the NS1 subunit from 3F5T. Molecular interactions between 3F5T and the p85β i-SH2 domain subunit of 3L4Q were visualized.

### 2.4. Plasmids and Site-Directed Mutagenesis

Plasmid pBudCE4.1 (Invitrogen) co-expressing A/Duck/Hubei/L-1/2004 [H5N1] NS1 complementary DNA (cDNA; HA-tagged) and green fluorescent protein (GFP) was generated as previously described [9]. Plasmid encoding A/Grey Heron/Hong Kong/837/2004 [H5N1] *NS* gene was provided by Dr. Leo L.M. Poon (University of Hong Kong, Hong Kong). Plasmids (pLLB) [32] encoding the eight A/WSN/33 [H1N1] gene segments (*HA*, *NA*, *NP*, *NS*, *PA*, *PB1*, *PB2*, *M*) were provided by Dr. Earl G. Brown (University of Ottawa, Ottawa, ON, Canada). The A/Grey Heron/Hong Kong/837/2004 [H5N1] *NS* gene was cloned into the pLLB plasmid using homologous recombination as described previously [32]. Site-directed mutagenesis was performed to introduce a Y84F mutation in pBudCE4.1-NS1-HA-GFP and pLLB-A/Grey Heron/Hong Kong/837/2004 [H5N1]-NS using the QuikChange Site-Directed Mutagenesis Kit and XL1-Blue supercompetent cells purchased from Agilent Technologies (Santa Clara, CA, USA) following the manufacturer’s protocol. Complimentary oligonucleotide primers (forward 5′GCCGGCTTCACGCTTCCTAACTGACATGAC3′, reverse 5′GTCATGTCAGTTAGGAAGCG TGAAGCCGGC3′) containing the desired Y84F mutation were synthesized by ACGT Corporation (Toronto, ON, Canada). The resulting pBudCE4.1-NS1-Y84F-HA-GFP plasmid and pLLB-A/Grey Heron/Hong Kong/837/2004 [H5N1] *NS*-Y84F gene were sequenced by ACGT Corporation to confirm the Y84F mutation.

### 2.5. Transfections

HeLa cells were seeded in 6-well plates at 2 × 10^5^ cells/well in 2 mL 10% FCS DMEM and incubated at 37 °C in 5% CO_2_ for 24 hours (h). Cells were transfected with 1.25 μg/well of pBudCE4.1-GFP (vector), pBudCE4.1-NS1-HA-GFP (NS1-wt), or pBudCE4.1-NS1-Y84F-HA-GFP (NS1-Y84F) using Lipofectamine™ LTX Reagent (Invitrogen) following the manufacturer’s protocol and as previously described [9].

### 2.6. Western Immunoblots

Transfected HeLa cells were either left untreated or treated with 1 × 10^3^ U/mL IFN-β-1a for 15 minutes (min) at 37 °C. Cells were lysed on ice using lysis buffer containing 1% Triton X-100, 0.5% NP-40, 150 mM NaCl, 10 mM Tris [pH 7.4], 1 mM EDTA, 1 mM EGTA, 0.2 mM Na_3_VO_4_, 0.2 mM PMSF, 10 μg/mL Aprotinin, 2 μg/mL Pepstatin A, and 1 mM Na_4_P_2_O_7_. An amount of 25 μg of each sample lysate was used for Western immunoblots. Sample lysates were denatured in 5× sample reducing buffer and resolved by SDS-PAGE. Proteins were transferred onto a nitrocellulose membrane and blocked with 5% BSA TBS-0.1% Tween-20 (TBS-T) for 1 h at room temperature. Membranes were probed with primary antibodies at a 1:1000 dilution in TBS-T overnight at 4 °C and secondary antibodies at a 1:10,000 dilution in TBS-T for 1 h at room temperature. Immunoblots were developed and proteins were visualized using SuperSignal West Pico Chemiluminescent Substrate Kit (Thermo Scientific, Waltham, MA, USA) following the manufacturer’s protocol. Band intensities were quantitated by densitometry using ImageJ software (National Institutes of Health, Bethesda, MD, USA).

### 2.7. Reverse Genetics

The 5 × 10^5^ HEK293T cells were transfected using Lipofectamine 2000 (Invitrogen) following the manufacturer’s protocol. Twenty-four hours before transfection, HEK293T cells were seeded in 6-well plates coated with poly-D-lysine (Sigma-Aldrich). An amount of 1 μg of each pLLB plasmid encoding one of the A/WSN/33 [H1N1] gene segments (*HA*, *NA*, *NP*, *NS*, *PA*, *PB1*, *PB2*, *M*) was transfected into the HEK293T cells to generate wildtype rA/WSN/33 virus as previously described [33]. pLLB-A/Grey Heron/Hong Kong/837/2004 [H5N1] *NS* and pLLB-A/Grey Heron/Hong Kong/837/2004 [H5N1] *NS*-Y84F were used in place of pLLB-A/WSN/33 [H1N1] *NS* to generate rWSN-GH-NS1-wt and rWSN-GH-NS1-Y84F respectively. Sixteen hours post-transfection, medium was replaced with 0% FCS DMEM containing 1 μg/mL tosyl phenylalanyl chloromethyl ketone (TPCK)-treated trypsin (Sigma-Aldrich). Forty-eight hours post-transfection, medium containing viral progeny was overlayed onto a monolayer of MDCK cells for 72 h. Viral yield was determined by plaque assay.

### 2.8. Virus Infection

#### 2.8.1. In Vitro

The 2 × 10^5^ A549 cells, STAT1^+/+^ and STAT1^−/−^ MEFs were seeded in 24-well plates for 24 h and then washed twice with phosphate buffer solution (PBS) and infected in triplicate with each of the rA/WSN/33 viruses at a multiplicity of infection (MOI) of 0.01 in the presence of 0.5 μg/mL (MEFs) or 1 μg/mL (A549) TPCK-treated trypsin. Medium was collected at the indicated times post-infection and viral titers were determined by plaque assay in MDCK cells.

#### 2.8.2. In Vivo

C57BL/6 mice 8–10 weeks of age were anesthetized by intraperitoneal injection with ketamine (Ketalean, Bimeda, Cambridge, ON, Canada) and xylazine (Rompun, Bayer, Mississauga, ON, Canada), and infected intranasally with 1 × 10^5^ plaque-forming units (PFU) of rA/WSN/33-GH-NS1-wt, or rA/WSN/33-GH-NS1-Y84F diluted in 50 μL of PBS. Infected mice were monitored daily for weight-loss and sacrificed by cervical dislocation on days 1 and 3 post-infection. Lungs from infected mice (*n* = 5) were harvested, weighed, and stored at −80 °C. The lungs were then thawed and mechanically homogenized on ice in 500 μL of serum-free DMEM containing 1 μg/mL TPCK-treated trypsin. The homogenized lung tissues were centrifuged at 12,000× *g* and the supernatants were used to determine lung viral titers by plaque assay.

Lungs were also harvested for flow cytometry analysis of neutrophil infiltration (*n* = 5). Lungs were perfused by slowly injecting 10 mL of PBS into the right ventricle of the heart. The lungs were mashed and incubated at 37 °C for 30 min in the presence of 1 mM CaCl_2_, 1.8 mM MgCl_2_, 1 mg/mL collagenase D (Roche, Penzberg, Germany) and 1 mg/mL DNase I (Thermo Scientific). Isolated cells were passed through a 70 μm cell strainer to obtain a single-cell suspension and red blood cells were lysed using ammonium-chloride-potassium (ACK) lysing buffer (150 mM NH_4_Cl, 10 mM KHCO_3_, and 0.1 mM Na_2_EDTA) for 5 min on ice. Cells were counted using a hemocytometer. Additional lungs were harvested on days 1 (wt, *n* = 5; Y84F, *n* = 4) and 3 (wt, *n* = 5; Y84F, *n* = 3) post-infection for histology. Lungs were placed into embedding cassettes and fixed using 4% formalin-PBS (Sigma-Aldrich) and stored at 4 °C.

#### 2.8.3. IFN-β Treatment In Vivo

Infected C57BL/6 mice were treated with 1× PBS or 1 × 10^5^ U of murine IFN-β1 diluted in 1× PBS by intraperitoneal injection at 8 h post-infection.

### 2.9. Plaque Assay

The 5 × 10^5^ MDCK cells were seeded in 6-well plates for 24 h until they formed an 80% confluent monolayer. Samples containing rIAVs were serially diluted in serum-free DMEM containing 1 μg/mL TPCK-trypsin. MDCK cells were washed twice with PBS and infected with 800 μL of the serially diluted rIAVs. Infected MDCK cells were incubated at 37 °C for 1 h to allow virus adsorption. An amount of 2 mL of 0.65% agarose diluted in serum-free DMEM in the presence of 1 μg/mL TPCK-trypsin was then overlaid onto the infected MDCK cells. MDCK cells were incubated at 37 °C for 72 h, then fixed using a 3:1 methanol:acetic acid solution. Plaques were enumerated to determine the viral titer, recorded as the number of PFU/mL of medium or PFU/g of lung tissue.

### 2.10. RNA Extraction and cDNA Synthesis

RNA was extracted and purified from infected A549 cells and the homogenized lung tissues of infected mice using the RNeasy Mini Kit (Qiagen, Venlo, The Netherlands), according to the manufacturer’s protocol. cDNAs were synthesized using 0.5 μg/sample of RNA, random primers, and M-MLV reverse transcriptase (Invitrogen), following the manufacturer’s protocol. cDNAs were also synthesized from 1 μg of RNA purified from uninfected A549 cells and MEFs treated for 16 h with 1 × 10^3^ U/mL of human IFN-β-1a and 1 × 10^3^ U/mL of murine IFN-β1, respectively.

### 2.11. qPCR

Quantitative polymerase chain reaction (qPCR) was performed using the LightCycler FastStart DNA Master SYBR Green PLUS I kit (Roche) and a LightCycler (Roche), following the manufacturer’s protocol as described previously [34]. Primers for target IFN stimulated genes (ISGs; Table 1) were synthesized by ACGT Corporation. Standard curves for each gene were generated using cDNAs from uninfected A549 cells and MEFs treated with 1 × 10^3^ U/mL of human IFN-β-1a and 1 × 10^3^ U/mL of murine IFN-β1, respectively. qPCR data were analyzed using LightCycler Data Analysis Software (Roche).

### 2.12. IFN-β ELISA

IFN-β production in the lungs of rIAV-infected C57BL/6 mice and by rIAV-infected A549 cells was quantified using the Legend Max ELISA kit (BioLegend) and the Verikine IFN-β enzyme-linked immunosorbent assay (ELISA) kit (PBL Assay Science), respectively, following the manufacturers’ protocols. Culture medium and homogenized lung supernatants—diluted with 500 µL DMEM—containing viral progeny, were stored at −80 °C prior to use.

### 2.13. FACS Analysis of IFNAR1 and IFNAR2 Expression

Twenty-four hours post-transfection, HeLa cells were harvested using Versene (Gibco, Waltham, MA, USA). Cells were washed with fluorescence-activated cell sorting (FACS) buffer (2% FCS in PBS) and resuspended in 200 μL of FACS buffer containing anti-human IFNAR1 or anti-human IFNAR2 at a 1:100 dilution for 45 min on ice. Cells were then washed three times and resuspended in 200 μL of FACS buffer containing anti-mouse IgG (Alexa Fluor 647) at a 1:100 dilution for 30 min on ice. Untransfected and transfected HeLa cells incubated with anti-mouse IgG (Alexa Fluor 647) alone were used as controls. Flow cytometry was performed using a FACSCalibur (BD Biosciences, San Jose, CA, USA) and data were analyzed using FlowJo software (FlowJo, Ashland, OR, USA). Cells were gated based on GFP expression.

### 2.14. Histology and Identification of Lung Neutrophils

Harvested lungs were embedded in paraffin and 5 μm thin sections containing multiple lobes were mounted onto slides and stained with hematoxylin and eosin (H&E). Sections were scanned using an Aperio ScanScope XT slide scanner (Leica Biosystems, Wetzlar, Germany) at 20× magnification and images were analyzed using Aperio ImageScope software (Leica Biosystems).

Single cell suspensions were prepared from lung aspirates and cells were blocked with mouse serum (Sigma-Aldrich) for 15 min on ice prior to staining. The 5 × 10^5^ cells/sample were stained with antibodies specific for mouse CD45, CD11b and Ly6G, or the appropriate isotype control antibodies for 45 min on ice. Compensations were conducted using anti-rat/hamster Ig, *κ* beads (BD Biosciences) and isotype control antibodies. Flow cytometry was performed using a LSR II (BD Biosciences) and data were analyzed using FlowJo software (FlowJo).

### 2.15. Statistical Analyses

An unpaired Student’s *t*-test was used to analyze differences among groups. A paired Student’s *t*-test was used to analyze differences among groups where *n* represents the same treatment from three independent experiments. *p*-values < 0.05 were considered statistically significant (* *p* < 0.05, ** *p* < 0.01, and *** *p* < 0.001).

## 3. Results

### 3.1. A Y84F Mutation in the H5N1 NS1 Conserved Putative SH2-Binding Domain Affects the Ability for NS1 to Upregulate AKT Phosphorylation

In an earlier publication, we provided evidence that cells expressing the A/Duck/Hubei/2004/L-1 [H5N1] NS1 are less responsive to the antiviral effects of IFN, exhibiting reduced IFN-inducible STAT1, STAT2 and STAT3 phosphorylation, thereby affecting the downstream events associated with STAT activation [9]. We have extended our studies to investigate the mechanism(s) whereby NS1 invokes these effects. Phosphorylation-independent binding of H1N1 NS1 to the p85β subunit of PI3K results in the phosphorylation of AKT, mediated by the catalytic activity of the p110 subunit, thereby enhancing viral replication [25,26]. This NS1-p85β binding has been ascribed to an SH2-binding domain in NS1, since a tyrosine to phenylalanine mutation at residue 89 (Y89F), within this domain, abrogated NS1 binding to host cell p85β and reduced IAV replication [25,26,28]. Notably, a number of IFN-inducible signaling effectors have SH2 domains, including STATs, from which we infer that a similar mechanism of NS1 binding to host transcription factors or signaling effectors may reduce IFN-inducible responses.

**Figure 1:**
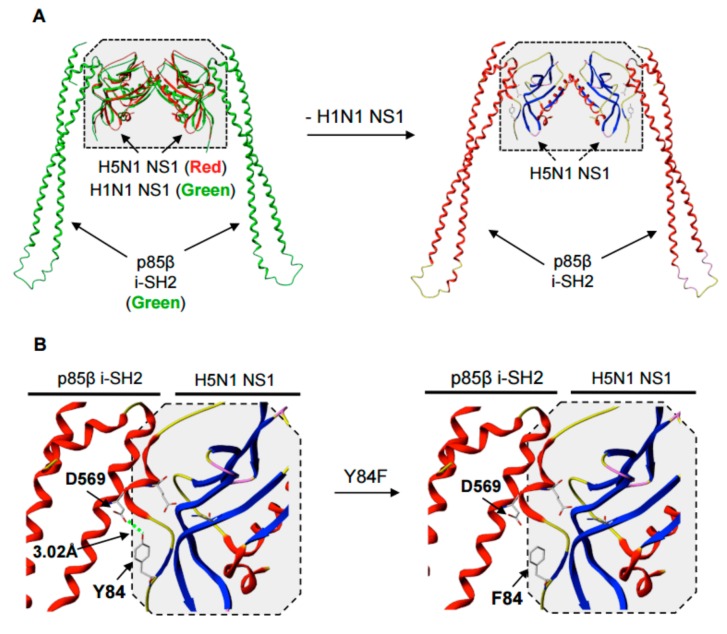
In silico modeling of the Y84F (tyrosine to phenylalanine) mutation in the putative Src homology 2 (SH2)-binding domain of A/Duck/Hubei/L-1/2004 [H5N1] non-structural protein 1 (NS1). (**A**) Ribbon diagrams of the A/Vietnam/1203/2004 [H5N1] H5N1 NS1 (PDB: 3F5T) and p85β complex, based on a crystallized structure of the H1N1 NS1 and p85β internal SH2 (i-SH2) domain complex (PDB: 3L4Q). (**B**) Ribbon diagrams showing the effect of the Y84F mutation on NS1-p85β binding.

The A/Duck/Hubei/L-1/2004 (H5N1) NS1 is evolutionarily distinct from both A/Puerto Rico/8/34 [H1N1] and A/WSN/33 [H1N1] NS1 proteins, and contains a five amino acid deletion at residues 80–84. Due to these differences in the NS1 amino acid sequences, we generated an in silico model of the H5N1 NS1-p85β i-SH2 interaction, using published crystallized structures of an H1N1 NS1 and p85β i-SH2 complex (PDB: 3L4Q, green) [31] and an H5N1 NS1 (A/Vietnam/1203/2004) containing the same five amino acid deletion (PDB: 3F5T, red) [15] (Figure 1A). This in silico model shows that residue Y84 in the H5N1 NS1 putative SH2-binding domain may interact via hydrogen bonding with residue D569 in the p85β i-SH2 domain and that a Y84F substitution eliminates this interaction (Figure 1B).

**Figure 2:**
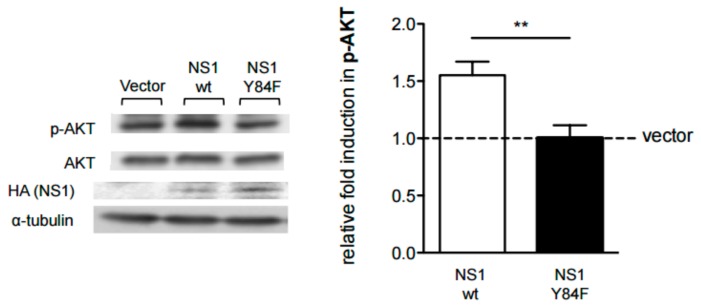
H5N1 NS1-Y84F is unable to upregulate protein kinase B (AKT) phosphorylation. HeLa cells were transfected with vector alone, vector carrying the NS1-wt complementary DNA (cDNA) (□), or NS1-Y84F cDNA (■). 24 hours (h) post-transfection, cells were lysed and lysates were resolved by SDS-PAGE and immunoblotted with an anti-phospho (p)-AKT (Ser473) antibody. The blot was then stripped and re-probed with an antibody against AKT. A separate aliquot of the same cell lysate was resolved by SDS-PAGE and immunoblotted with antibodies against HA (NS1) and α-tubulin. Band intensities were quantitated and the relative induction in p-AKT was determined, normalizing to AKT. Data are presented as the mean +/− standard error (SE) and are representative of three independent experiments. ** *p* < 0.01.

Accordingly, we used site-directed mutagenesis to introduce the Y84F mutation within the conserved H5N1 NS1 putative SH2-binding domain, to examine its contribution to NS1-mediated down-regulation of IFN-inducible STAT phosphorylation and IAV virulence. In a first series of experiments, we examined the effects of expression of the wildtype NS1 (NS1-wt) or Y84F mutation (NS1-Y84F) in HeLa cells on AKT phosphorylation, a signaling effector downstream of PI3K. The objective was to demonstrate that, in contrast to NS1-wt, which is known to induce AKT phosphorylation, NS1-Y84F would fail to increase AKT phosphorylation. As anticipated, the results in Figure 2 reveal that 24 h post-transfection, cells expressing NS1-wt exhibit a 1.5-fold increase (significant *p* < 0.01) in AKT phosphorylation compared with cells expressing NS1-Y84F.

### 3.2. A Y84F Mutation Abrogates NS1-Mediated Inhibition of Type I IFN Signaling

**Figure 3:**
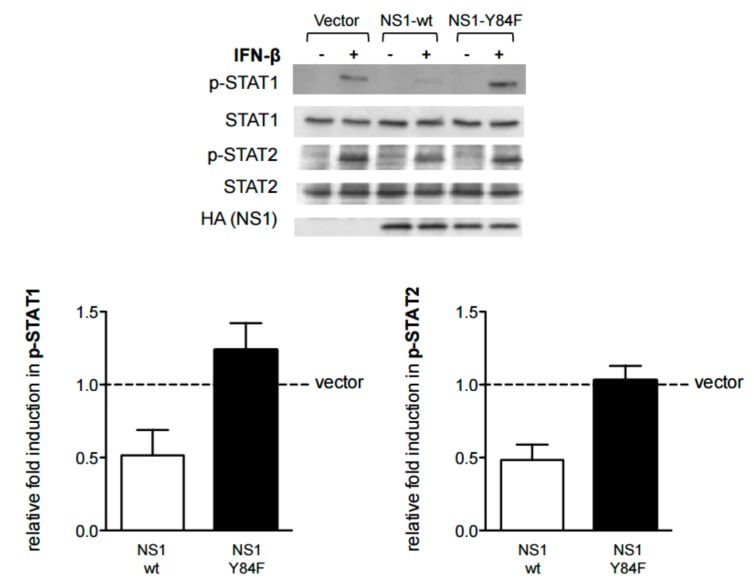
The Y84F mutation abrogates H5N1 NS1-mediated inhibition of interferon (IFN)-inducible signal transducer and activator of transcription (STAT) phosphorylation. HeLa cells were transfected with vector alone, or vector carrying the NS1-wt cDNA (□), or NS1-Y84F cDNA (■). 24 h post-transfection, cells were either left untreated or treated with 1000 U/mL of IFN-β for 15 minutes (min). Cells were lysed, lysates resolved by SDS-PAGE and immunoblotted with antibodies against p-STAT (Tyr701), p-STAT2 (Tyr690), or HA (NS1). Blots were then stripped and re-probed with antibodies against STAT1 or STAT2. Band intensities were quantitated and the relative induction in p-STAT1 and p-STAT2 was determined, normalizing to STAT1 and STAT2, respectively. Data are presented as the mean +/− SE and are representative of three independent experiments.

**Figure 4:**
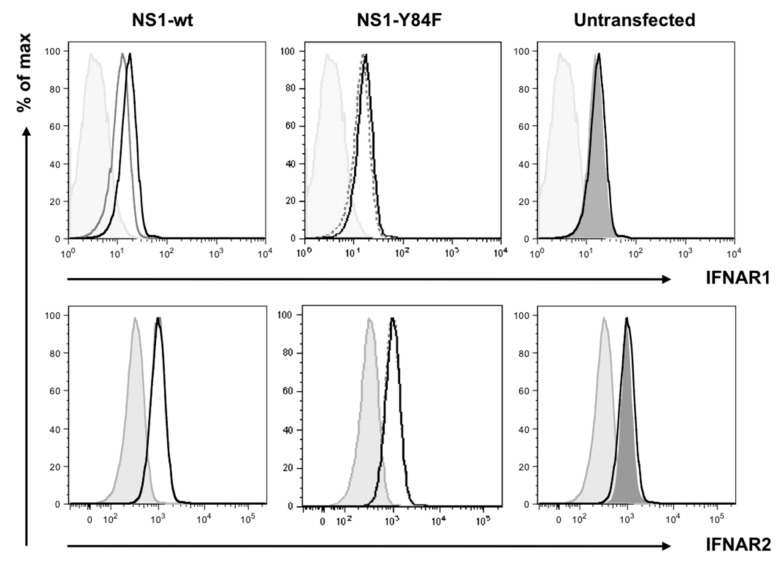
NS1-Y84F does not affect IFN-α/β receptor subunit (IFNAR) 1 expression. HeLa cells were transfected with vector alone (▬, black), vector carrying the NS1-wt cDNA (▬, grey), or NS1-Y84F cDNA (---). 24 h later, cells were stained with antibodies against IFNAR1 or IFNAR2. Transfected cells stained with the Alexa Fluor 647 secondary antibody alone served as the isotype control (▬, light grey fill). Untransfected HeLa cells (no line, dark gray fill) were also stained. Cells were analyzed using a FACSCalibur, gating on green fluorescent protein (GFP)+ cells, then analyzing for IFNAR1 or IFNAR2 expression. Data are representative of two independent experiments.

Next, we performed a series of experiments to examine the effects of the Y84F mutation on the ability of NS1 to regulate the type I IFN signaling response. As mentioned, we have shown that H5N1 NS1 expression in HeLa cells inhibits IFN-inducible STAT1 and STAT2 phosphorylation [9]. Here, we show that the levels of IFN-inducible STAT1 and STAT2 phosphorylation are unaffected in cells expressing the mutant NS1-Y84F, compared with a reduction in cells expressing NS1-wt (Figure 3). Having demonstrated that expression of NS1-wt reduces cell surface IFNAR1 expression [9], we likewise examined whether the NS1-Y84F mutant would affect IFNAR1 cell surface expression. The data in Figure 4 reveal that in contrast to NS1-wt expression, which reduces IFNAR1 but not IFNAR2 expression, NS1-Y84F expression has no effect on IFNAR1 or IFNAR2 expression. The reduction in surface IFNAR1 expression in cells transfected with NS1-wt is similar in magnitude to the reduction observed in cells which have been treated with IFN-β (data not shown).

### 3.3. Effects of the Conserved Putative SH2-Binding Domain in NS1 on Virus Replication

**Figure 5:**
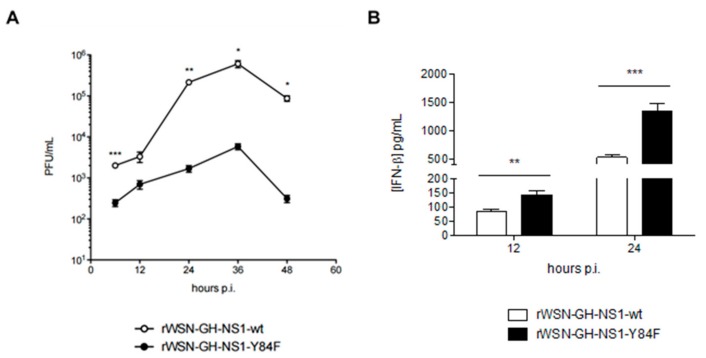
The Y84F mutation inhibits recombinant IAV (rIAV) [H1N1] replication and enhances IFN-β production in human A549 lung epithelial cells. (**A**) A549 cells were infected with rWSN-GH-NS1-wt (○) or rWSN-GH-NS1-Y84F (●) at a multiplicity of infection (MOI) of 0.01. Culture supernatants from rWSN-GH-NS1-wt (□) or rWSN-GH-NS1-Y84F (■) infected cells were collected at 6, 12, 24, 36, and 48 h post-infection and viral titers were determined by plaque assay in Madin-Darby canine kidney (MDCK) cells. (**B**) IFN-β levels were measured in the culture supernatants collected at 12 and 24 h post-infection by enzyme-linked immunosorbent assay (ELISA). Data are presented as the mean +/− SE and are representative of three (titration) and two (ELISA) independent experiments. * *p* < 0.05, ** *p* < 0.01, and *** *p* < 0.001.

To further examine the importance of this Y84 residue within the putative SH2-binding domain in H5N1 NS1, we generated rIAVs expressing either the H5N1 NS1-wt or NS1-Y84F: rWSN-GH-NS1-wt and rWSN-GH-NS1-Y84F, respectively. Time course studies in A549 human lung epithelial cells revealed that rWSN-GH-NS1-wt grows to approximately 100-fold higher titers than rWSN-GH-NS1-Y84F (Figure 5A). For both rIAVs, viral titers increase up to 36 h post-infection, then decline at 48 h. These data support earlier published data [28] that the conserved putative SH2-binding domain of H1N1 NS1 is important for IAV replication in vitro. In addition, A549 cells infected with rWSN-GH-NS1-Y84F produced approximately 1.7-fold and 2.6-fold more IFN-β than cells infected with rWSN-GH-NS1-wt.

**Figure 6:**
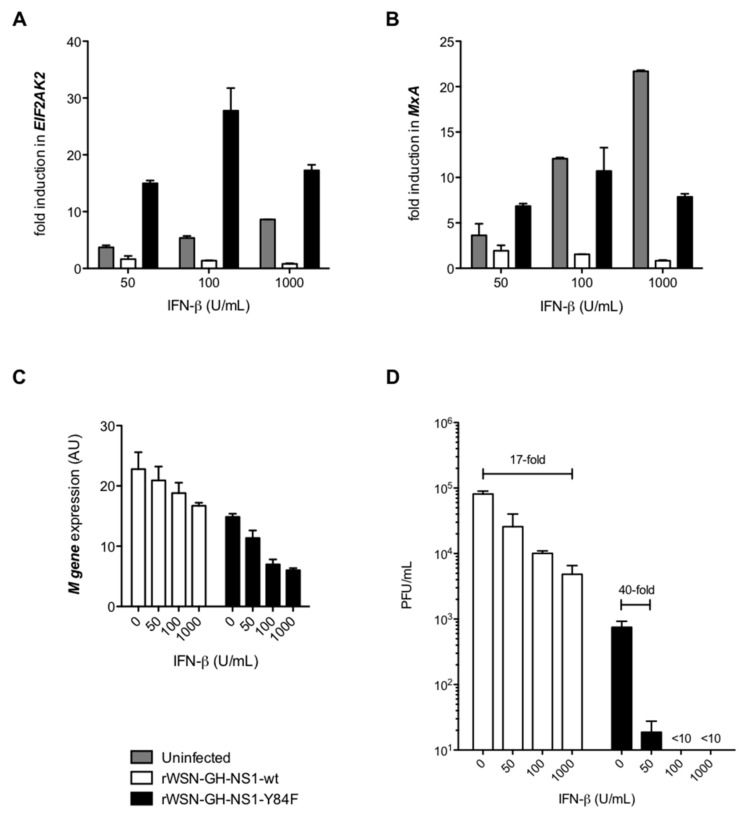
The Y84F mutation confers greater sensitivity to the antiviral effects of IFN-β. A549 cells were left uninfected (■) or infected with rWSN-GH-NS1-wt (□) or rWSN-GH-NS1-Y84F (■) at a MOI of 0.01. At 12 h post-infection, cells were either left untreated, or treated with 50, 100, or 1000 U/mL of IFN-β. RNA was purified from cells at 24 h post-infection (i.e., 12 h post-IFN-β treatment) and cDNA was synthesized. Quantitative polymerase chain reaction (qPCR) was performed to determine the relative expression of IFN-inducible (**A**) *EIF2AK2* and (**B**) *MxA,* normalized to *HPRT1*. Data are presented as the fold-induction of each IFN stimulated gene (ISG) relative to infected or uninfected A549 cells that were not treated with IFN-β. (**C**) IAV *M* gene expression was determined by qPCR at 48 h post-infection (36 h post-IFN-β treatment). (**D**) Viral titers in culture supernatants collected at 48 h post-infection (36 h post-IFN-β treatment) were determined by plaque assay in MDCK cells. Data are presented as the mean +/− SE and are representative of two independent experiments.

Next, we conducted a series of experiments to investigate whether, as we had observed for HeLa cells expressing NS1-wt or NS1-Y84F, an intact putative SH2-binding domain influences the response to IFN-β treatment. Specifically, A549 cells were infected with rWSN-GH-NS1-wt or rWSN-GH-NS1-Y84F at a MOI of 0.01 for 12 h, and then treated with varying doses of IFN-β. This 12 h time point post-infection was selected to allow for NS1-wt or NS1-Y84F to be expressed in infected cells, yet early enough in the infection to preclude profound differences in viral titers. IFN-β treatment of uninfected A549 cells resulted in the expected increases in gene expression for the ISGs *EIF2AK2* (Figure 6A) and *MxA* (Figure 6B), both implicated in mediating antiviral activity. At 12 h post-IFN-β-treatment, *EIF2AK2* (Figure 6A) and *MxA* (Figure 6B) were induced in cells infected with rWSN-GH-NS1-Y84F, whereas cells infected with rWSN-GH-NS1-wt exhibited no ISG induction.

In subsequent experiments, we examined viral replication in IFN-β treated and rIAV infected A549 cells 36 h after IFN-β treatment, i.e., 48 h post-infection. This time point was chosen to best represent the outcome of ISG induction on IAV replication. Following IFN-β treatment, we observed a greater reduction in *M* gene expression in A549 cells infected with rWSN-GH-NS1-Y84F, compared to cells infected with rWSN-GH-NS1-wt (Figure 6C). Our measurements of virus in culture supernatants revealed that IFN-β treatment for 36 h reduced viral titers in a dose-dependent manner, albeit to a greater extent in the cells infected with rWSN-GH-NS1-Y84F: 1000 U/mL of IFN-β reduced the viral titer of rWSN-GH-NS1-wt by 1-log (17-fold), in comparison to 50 U/mL of IFN-β that reduced the titer of rWSN-GH-NS1-Y84F by 1-log (40-fold; Figure 6D). Notably, IFN-β doses of 100 and 1000 U/mL reduced viral titers in rWSN-GH-NS1-Y84F infected A549s to <10 PFU/mL.

**Figure 7:**
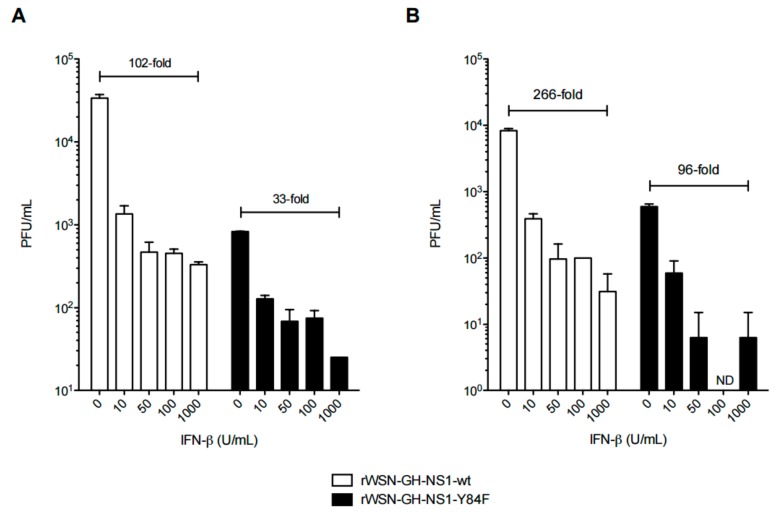
IFN-β pre-treatment inhibits rIAV replication. A549 cells were either left untreated or treated with 50, 100, or 1000 U/mL of IFN-β for 16 h and then infected with rWSN-GH-NS1-wt (□) or rWSN-GH-NS1-Y84F (■) at a MOI of 0.01. Culture supernatants were collected at (**A**) 24 and (**B**) 48 h post-infection (40 and 64 h post-IFN-β treatment, respectively) and viral titers were determined by plaque assay in MDCK cells. Data are presented as the mean +/− SE and are representative of two independent experiments. ND ~ not detected.

To determine whether pre-treatment of cells with IFN-β would override the inhibitory effects of NS1-wt, we treated A549s cells with increasing doses of IFN-β 16 h prior to infection with the rIAVs (i.e., to eliminate the inhibitory effects of NS1 by generating IFN-inducible antiviral responses that would precede infection). The results in Figure 7 show a greater than 1-log fold reduction in viral titers at 24 (Figure 7A) and 48 (Figure 7B) h post-infection, when cells are pre-treated with different doses of IFN-β, for both rIAVs, in comparison to untreated cells that are more susceptible to viral replication following infection with the rIAV expressing NS1-wt. Notably, pre-treatment of A549 cells with 10 U/mL of IFN-β prior to infection with rWSN-GH-NS1-wt was sufficient to reduce the viral titers at 24 and 48 h by 1-log fold.

**Figure 8:**
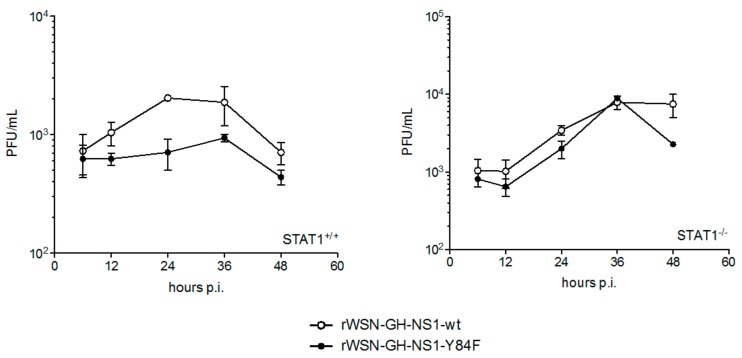
rWSN-GH-NS1-wt and rWSN-GH-NS1-Y84F replicate to higher titers in STAT1^−/−^ mouse embryonic fibroblasts (MEFs) in comparison to STAT1^+/+^ MEFs. STAT1^+/+^ and STAT1^−/−^ MEFs were infected with rWSN-GH-NS1-wt (○) or rWSN-GH-NS1-Y84F (●) at a MOI of 0.01. Culture supernatants were collected at 6, 12, 24, 36 and 48 h post-infection and viral titers were determined by plaque assay in MDCK cells. Data are presented as the mean +/− SE and are representative of two independent experiments.

In subsequent experiments, we infected STAT1^+/+^ and STAT1^−/−^ MEFs with the rIAVs to further determine whether the differences in viral replication that we observed between rWSN-GH-NS1-wt and rWSN-GH-NS1-Y84F were due primarily to effects of NS1 on the IFN-α/β response. STAT1^−/−^ mice and STAT1^−/−^ MEFs are unable to respond to IFN-α/β [35,36]. In STAT1^+/+^ MEFs, viral titers increased by approximately 2.6-fold for rWSN-GH-NS1-wt between 6 and 36 h post-infection, whereas a marginal 1.5-fold increase in viral titers was observed for rWSN-GH-NS1-Y84F. In contrast, both rIAVs replicated in STAT1^−/−^ MEFs with rWSN-GH-NS1-wt and rWSN-GH-NS1-Y84F viral titers increasing by approximately 7.6- and 10.9-fold, respectively (Figure 8).

### 3.4. The Conserved Putative SH2-Binding Domain in NS1 Contributes to IAV Virulence In Vivo, Affecting the IFN Response

In a final series of experiments, we examined the effects of the conserved putative SH2-binding domain in NS1 on IAV virulence in vivo, in the context of the IFN response. Studies were conducted in C57BL/6 mice to compare the infectivity of rWSN-GH-NS1-wt with rWSN-GH-NS1-Y84F, following intranasal inoculation. Initial readouts for infectivity were weight loss and lung viral loads on days 1 and 3 post-infection.

**Figure 9:**
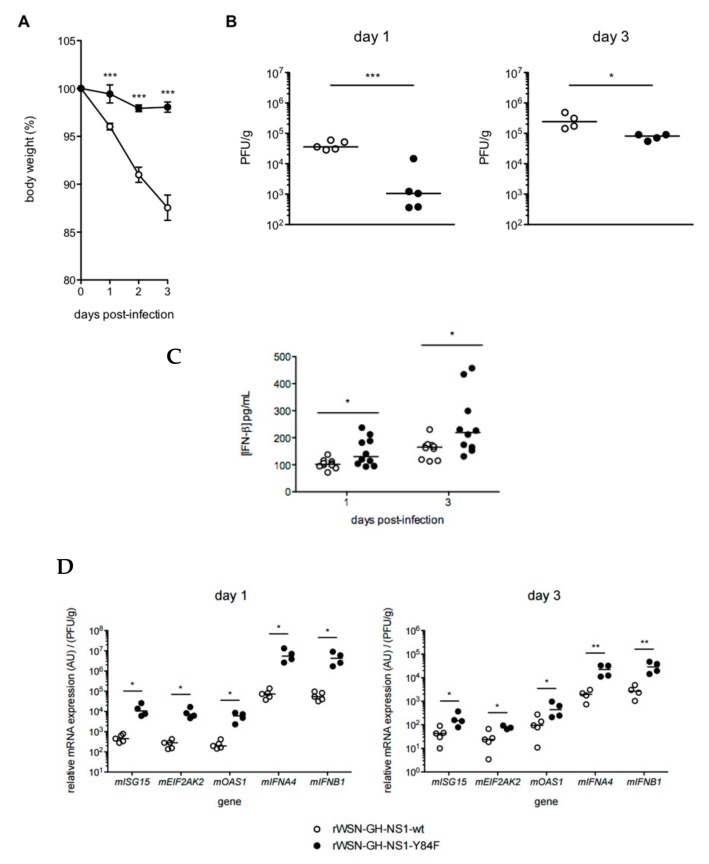
Mice infected with rIAV expressing the Y84F mutant NS1 experience delayed weight-loss and lower lung viral titers on day 1 and day 3 post-infection. C57BL/6 mice were infected with 1 × 10^5^ plaque-forming units (PFU) of rWSN-GH-NS1-wt (○) or rWSN-GH-NS1-Y84F (●) by intranasal inoculation. (**A**) Weight-loss was monitored up to day 3 post-infection. Mice were euthanized on days 1 and 3 post-infection and (**B**) lung viral titers were determined by plaque assay in MDCK cells and (**C**) IFN-β production in the lungs was determined by ELISA. (**D**) RNA was purified from the lung tissues of rIAV-infected mice on days 1 and 3 post-infection and cDNA was synthesized. qPCR was performed to determine the relative expression of murine *ISG15*, *EIF2AK2*, *OAS1*, *IFNA4*, and *IFNB1*, normalized to *HPRT1* and lung viral titer. Data are representative of three independent experiments. * *p* < 0.05, ** *p* < 0.01, and *** *p* < 0.001.

C57BL/6 mice received an intranasal inoculation of 1 × 10^5^ PFU of either the rIAV expressing the NS1-wt or the rIAV expressing the NS1-Y84F. Weight loss and lung ISG expression were recorded on days 1 and 3 post-infection (Figure 9). Mice infected with virus expressing the mutant NS1-Y84F lost less than 5% of their starting body weight by day 3 post-infection, whereas mice infected with virus expressing the NS1-wt lost greater than 10% of their starting body weight over the same time period (Figure 9A). Furthermore, mice infected with virus expressing NS1-wt had higher lung viral titers than mice infected with virus expressing NS1-Y84F on days 1 and 3 post-infection (Figure 9B).

Viral titers increased for both rIAVs expressing NS1-wt and NS1-Y84F between days 1 and 3 post-infection. To investigate whether the IFN response contributed to these differences in lung viral titers, IFN-β production and ISG expression were examined in the lungs of mice on days 1 and 3 post-infection. In comparison to rWSN-GH-NS1-wt infected mice, we show that IFN-β production is elevated in the lungs of rWSN-GH-NS1-Y84F infected mice on both days 1 and 3 post-infection (Figure 9C). Lung ISG expression, which included expression levels for IFN-α4 and IFN-β, was considered a measure of the IFN response to infection. We observed approximately 0.5 to 1-log fold greater expression of *ISG15*, *EIF2AK2*, *OAS1*, *IFNA4*, and *IFNB1* in the lungs of rWSN-GH-NS1-Y84F infected mice on days 1 and 3 post-infection compared with the lungs of rWSN-GH-NS1-wt infected mice (Figure 9D).

**Figure 10:**
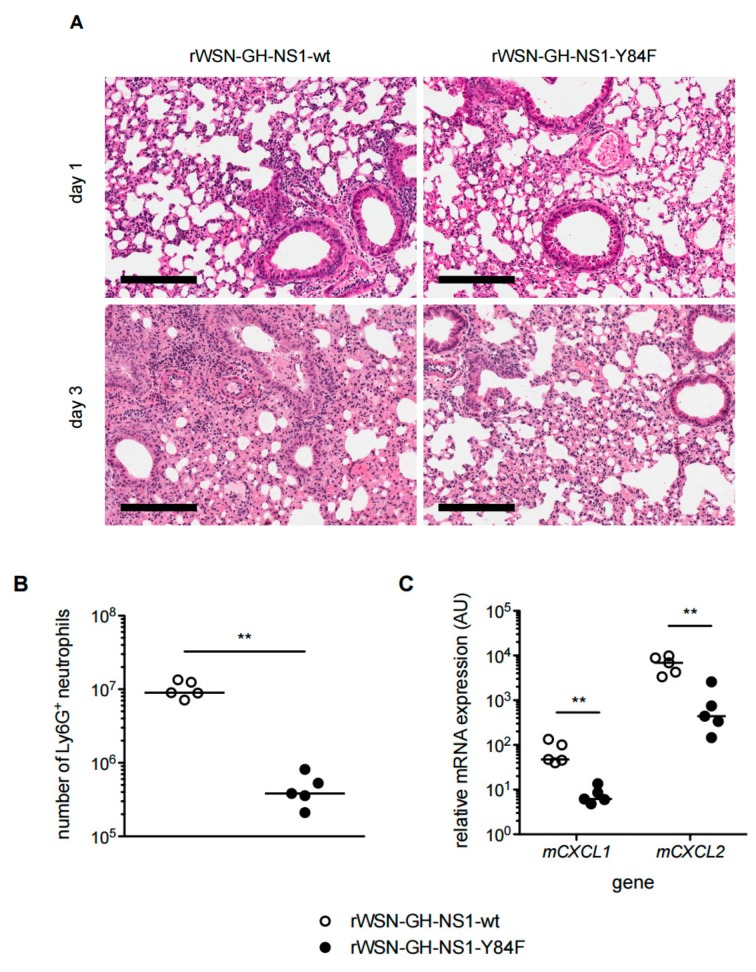
Mice infected with rIAV expressing the Y84F mutant NS1 experience reduced lung pathology and neutrophil infiltration early in infection. C57BL/6 mice were infected with 1 × 10^5^ PFU of rWSN-GH-NS1-wt (○) or rWSN-GH-NS1-Y84F (●) by intranasal inoculation. (**A**) Lungs were harvested on days 1 and 3 post-infection and processed to prepare thin tissue sections (5 μm) for H&E staining. Black bar = 200 μm. (**B**) Lungs were harvested on day 1 post-infection and perfused with PBS. Lung tissues were mashed mechanically and ammonium-chloride-potassium (ACK) lysis was performed to remove red blood cells. Neutrophils were quantified by flow cytometry with antibodies targeting CD45, CD11b, and Ly6G. (**C**) Lungs were harvested on day 1 post-infection, RNA was extracted, and cDNA synthesized. qPCR was performed to determine the relative expression of murine *CXCL1* and *CXCL2,* normalized to *HPRT1*. Data are representative of two independent experiments. ** *p* < 0.01.

Lung histology and H&E staining revealed more cell infiltrates in the lungs of rWSN-GH-NS1-wt infected mice compared with mice infected with rWSN-GH-NS1-Y84F (Figure 10A). Flow cytometry analysis of lung infiltrates on day 1 post-infection showed that there were of the order of 1-log fold more neutrophils (CD45+, CD11b+, Ly6G+) in the lungs of rWSN-GH-NS1-wt infected mice compared with the lungs from mice infected with rWSN-GH-NS1-Y84F (Figure 10B). Consistent with greater neutrophil numbers, mice infected with virus expressing NS1-wt exhibited 7.5-fold and 15-fold greater *CXCL1* and *CXCL2* gene expression, respectively, in their lungs compared with mice infected with virus expressing NS1-Y84F on day 1 post-infection (Figure 10C). CXCL1 and CXCL2 are the major chemoattractants responsible for recruiting neutrophils.

**Figure 11:**
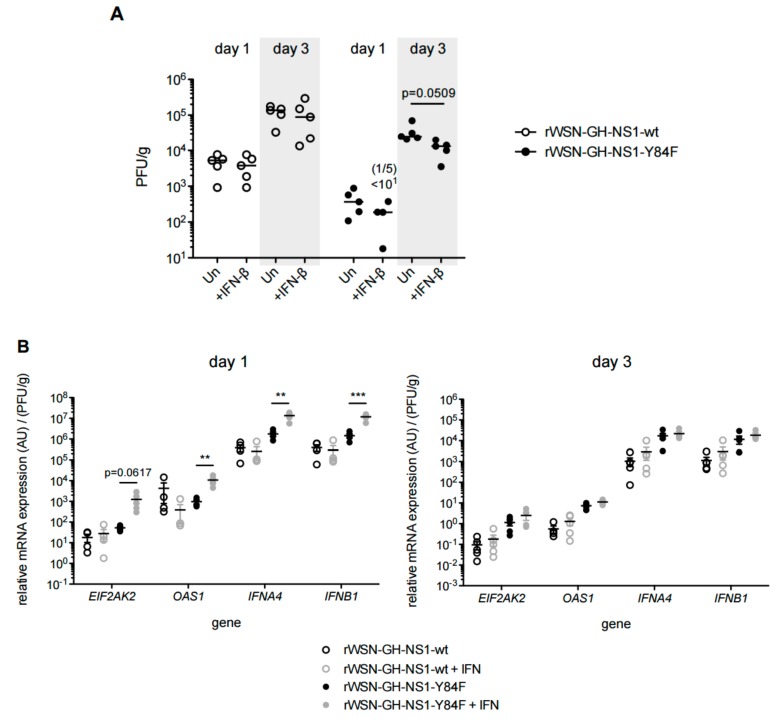
Mice infected with rIAV expressing the Y84F mutant NS1 are more sensitive to the antiviral effects of IFN-β. C57BL/6 mice were infected with 1 × 10^5^ PFU of rWSN-GH-NS1-wt (○) or rWSN-GH-NS1-Y84F (●) by intranasal inoculation and injected by the intraperitoneal route with PBS or 1 × 10^5^ U of murine IFN-β1 at 8 h post-infection. (**A**) Lung viral titers were determined on day 1 and 3 post-infection by plaque assay in MDCK cells. On day 1 post-infection, one of the five mice in the Y84F-infected and IFN-treated cohort, did not have a detectable lung viral titer (titer < 10^1^ PFU/mL). (**B**) RNA was purified from the lung tissues of rIAV infected and PBS or murine IFN-β1 treated mice on days 1 and 3 post-infection. cDNA was synthesized and qPCR was performed to determine the relative expression of murine *EIF2AK2*, *OAS1*, *IFNA4*, and *IFNB1*, normalized to *HPRT1* and lung viral titer. Data are representative of two independent experiments. ** *p* < 0.01, and *** *p* < 0.001.

To examine the potential differential effects of IFN treatment on viral replication following infection with the different recombinant viruses, mice were infected with virus expressing the NS1-wt or virus expressing the NS1-Y84F mutant and at 8 h post-infection mice were treated with a single dose of 1 × 10^5^ U of murine IFN-β1. While IFN-β treatment did not alter the lung viral titers of mice infected with rWSN-GH-NS1-wt, we observed a marginal reduction in the lung viral titers of mice infected with rWSN-GH-NS1-Y84F on days 1 and 3 post-infection (Figure 11A). Additionally, we observed increases in the expression of *EIF2AK2* (24-fold), *OAS1* (10-fold), *IFNA4* (10-fold) and *IFNB1* (10-fold) in the lungs of rWSN-GH-NS1-Y84F infected mice treated with IFN-β on day 1 post-infection compared with the lungs of infected, but untreated mice (Figure 11B). In contrast, there was no difference in the relative expression of these ISGs in the lungs of mice infected with rWSN-GH-NS1-wt, with or without IFN-β treatment.

## 4. Discussion

Viruses have evolved to encode non-structural proteins in their genomes that interact with host factors to enable viral replication. Moreover, there is accumulating evidence for virus–host protein–protein interactions mediated by SH2 binding: binding of IAV NS1 to the i-SH2 domain of p85β to activate PI3K signaling to enhance viral replication [25,26]; the Nef protein of human immunodeficiency virus (HIV)-1 is critical for high titer viral replication and its function is dependent on interactions with the Src family kinase, Hck, stabilized by SH2 binding interactions [37]; the Epstein–Barr virus latency-associated membrane protein, LMP2A, interacts with the signaling scaffold, Shb, mediated by SH2 domain interactions to activate AKT [38]; in silico studies have suggested a molecular model for STAT3 and STAT6 SH2 interactions with the g2-Herpesvirus saimiri Tip protein [39].

Notably, many of the virulence factors encoded by viruses target an IFN response, specifically by binding to and inhibiting the activities of STATs, thereby preventing the induction of an antiviral state. Binding of the measles virus P protein to the SH2 domain of STAT1 limits an IFN response to infection [40]. Other examples include the Nipah virus V, P, and W proteins [41], Sendai virus C protein [42], and hepatitis C virus (HCV) core protein [43,44]. Furthermore, binding of these viral proteins to the host protein SH2-binding domain is often tyrosine phosphorylation-independent, e.g., HIV-1 Nef protein and hepatitis C virus core protein SH2-binding interactions. Our earlier studies revealed that expression of avian H5N1 NS1 in HeLa cells led to a block in IFN signaling [9]. H5N1 NS1 reduced IFN-inducible tyrosine phosphorylation of STAT1, STAT2 and STAT3 and inhibited the nuclear translocation of phospho-STAT2 and the formation of IFN-inducible STAT1:STAT1-, STAT1:STAT3- and STAT3:STAT3-DNA complexes. We attributed the inhibition of IFN-inducible STAT signaling by NS1 in HeLa cells to be a consequence, in part, of NS1-mediated inhibition of expression of the IFN receptor subunit, IFNAR1. In support of this NS1-mediated inhibition, we observed a reduction in expression of IFNAR1 in ex vivo human non-tumor lung tissues infected with H5N1 and H1N1 viruses. Indeed, studies by Zurney et al. [45] comparing cardiotropic reovirus infection of cardiac myocytes and cardiac fibroblasts identified a correlation between greater surface expression of IFNAR1 in cardiac fibroblasts and greater IFN-inducible STAT phosphorylation and induction of ISGs resulting in reduced infectivity [45].

Herein, we have extended our earlier studies to interrogate the mechanism of NS1-mediated inhibition of the IFN response we observed. Given the evidence for viral protein binding to SH2 domains in host proteins, and cognizant of the strictly conserved putative SH2-binding domain in NS1 contributing to IAV virulence, we focused our studies on this domain. Both A/Duck/Hubei/L-1/2004 and A/Grey Heron/Hong Kong/837/2004 H5N1 NS1 SH2-binding domains contain a conserved tyrosine residue at position 84. Highly pathogenic H5N1 IAVs that emerged after the year 2000 have a five amino acid deletion in the linker region between the NS1 dsRNA-binding domain and the protein-binding effector domain, where the conserved tyrosine residue has shifted from position 89 to 84 [15,46]. This five amino acid deletion has been shown to increase the virulence of H5N1 IAVs [46], and based on our current studies, residue Y84 in the H5N1 NS1 putative SH2-binding domain is still important for the activation of AKT, similar to H1N1 NS1 proteins, which contain the conserved tyrosine residue at position 89 [25,26,28]. Our results suggest that host AKT-activation mediated by the NS1 putative SH2-binding domain is well conserved, highlighting its critical role in IAV replication. Indeed, close scrutiny of the adaptive mutations in H5N1 NS1 associated with increased virulence or host switching to mouse reveals that the putative SH2-binding domain and, more specifically the conserved tyrosine residue within this domain, is intact [47,48].

Beyond AKT activation, we show that the conserved putative SH2-binding domain in H5N1 NS1 influences the virulence of IAVs, specifically by limiting an IFN-induced antiviral response. Our data suggest that the inhibitory effects of this putative SH2-binding domain are associated with inhibition of IFN-induced STAT phosphorylation and subsequent ISG expression. Specifically, we provide evidence that, in contrast to cells expressing the intact NS1, cells expressing NS1 with a single Y84F mutation in this conserved putative SH2-binding domain are able to respond fully to IFN treatment in terms of STAT phosphorylation. It is noteworthy that our measure of IFN responsiveness, phosphorylation of STATs, occurs rapidly, within 15 min of IFN treatment. Additionally, cells expressing the Y84F mutant NS1 revert to normal IFNAR1 cell surface expression. We infer from these expression studies that the Y84 residue within NS1 is important for modulating host STAT phosphorylation and IFNAR1 expression, but further studies are required to determine the mechanism and whether NS1 induced PI3K activation may be involved in the observed phenotype. Moreover, studies to determine if NS1-mediated inhibition of IFN signaling is conserved in seasonal IAV strains, which do not encode a deletion in the linker region, may also be warranted.

These in vitro data provided the basis for generating recombinant H1N1 viruses (A/WSN/33 [H1N1]) encoding either the intact (rWSN-GH-NS1-wt) or Y84F mutant H5N1 NS1 (rWSN-GH-NS1-Y84F). In vitro, we showed that the virus expressing the intact H5N1 NS1 replicates to a greater extent in human lung epithelial cells than the virus expressing the SH2-binding domain mutant of NS1, which induced higher levels of IFN-β production. Indeed, IFNs-α/β are induced by IFN signaling in a positive feedback loop. In addition, cells infected with the recombinant virus expressing the mutant NS1 responded to IFN-β treatment with greater levels of ISG expression than cells infected with the virus expressing intact NS1, yet to a lesser extent than uninfected and IFN-β treated cells, in support of virus inhibiting the IFN response and that this is partially mediated by residue Y84 within the putative SH2-binding domain of NS1. The ISGs selected were based on their known antiviral properties: *EIF2AK2* encodes protein kinase RNA-activated (PKR), a host antiviral effector that inhibits cellular translation [49,50]. *OAS1* encodes 2′-5′-oligoadenylate synthetase 1, which activates RNase L in the presence of IAV viral RNA, resulting in viral and cellular RNA degradation [51]. In line with previous IAV replication studies performed in STAT1^+/+^ and STAT1^−/−^ MEFs [36], both rWSN-GH-NS1-wt and rWSN-GH-NS1-Y84F replicated poorly in STAT1^+/+^ MEFs in comparison to STAT1^−/−^ MEFs, thereby further highlighting a potential role for residue Y84 of NS1 in regulating the type I IFN response, and the importance of the host IFN response for limiting viral replication.

Extending these infection studies in vivo, we confirmed that the recombinant H1N1 virus expressing the H5N1 NS1 i-SH2-binding mutant replicated to lower levels than the virus expressing the intact NS1, the mice exhibiting a less severe course of disease, in terms of lung pathology and extent of lung infiltrating neutrophils. Gene expression levels for *CXCL1* and *CXCL2* were higher in the lungs of mice infected with the virus expressing intact NS1, consistent with their role in recruiting neutrophils. In the context of an IAV infection, neutrophils have been shown to play a role in lung tissue damage and increasing the severity of infection [52,53]. In addition, the IFN response to infection, in terms of IFN-β production and transcriptional induction of ISGs—including genes for IFN-α and IFN-β—were more extensive in the lungs of the mice infected with the virus expressing the mutant NS1 than in the lungs of the mice expressing the intact NS1. There is evidence also that CXCL1 and CXCL2, are down-regulated by IFN [54,55]. We speculate that the stronger IFN response in the mice infected with the virus expressing the mutant NS1 contributed to the reduced lung viral replication and less severe lung pathology. This is supported by our findings when mice were treated with IFN-β. Mice infected with virus expressing the mutant NS1 responded to IFN treatment with a more robust transcriptional induction of ISGs than mice infected with the virus expressing an intact NS1 on day 1 post-infection. This was reflected in the modest reduction in lung viral titers in IFN-β-treated mice infected with virus encoding the Y84F mutant NS1, which was not observed in mice infected with virus expressing an intact NS1.

These studies highlight the importance of an IFN response to the control of IAV infections and the potential for IFN treatment to be considered when there are outbreaks of highly virulent IAV strains. Additionally, these studies suggest that targeting the conserved tyrosine residue in the putative SH2-binding domain in IAV NS1 may be a therapeutic strategy in the absence of available vaccines. In ongoing studies, we are identifying additional host protein-binding partners that interact with and/or that are affected by Y84 within the NS1 putative SH2-binding domain in the context of an IFN response.

## 5. Conclusions

Our studies support a role for the strictly conserved residue, Y84, in a putative SH2-binding domain in H5N1 NS1 in inhibiting the host IFN antiviral response. Specifically, using rIAVs expressing a wildtype NS1 or NS1 encoding a Y84F mutation, we show in vitro and in vivo that targeting the conserved tyrosine residue inhibits virus replication and confers greater sensitivity to the antiviral effects of IFN.

## Figures and Tables

**Table 1:** List of human (*h*), mouse (*m*) and influenza A virus (*IAV*) qPCR primers used in this study.

Gene	Forward Primer (5′-3′)	Reverse Primer (5′-3′)
*(h) HPRT1*	TCCTCCTCTGCTCCGCCACC	TCACTAATCACGACGCCAGGGCT
*(h) MxA*	GATGATCAAAGGGATGTGGC	AGCTCGGCAACAGACTCTTC
*(h) EIF2AK2*	ACTTGGCCAAATCCACCTG	CCCAGATTTGACCTTCCTGA
*(m) HPRT1*	CATAACCTGGTTCATCATCGC	TCCTCCTCAGACCGCTTTT
*(m) ISG15*	CCCCAGCATCTTCACCTTTA	TGACTGTGAGAGCAAGCAGC
*(m) OAS1*	AGTTCTCCTCCACCTGCTCA	GGCTGTGGTACCCATGTTTT
*(m) EIF2AK2*	CTGTTGCAAGGCCAAAGTCT	GAACAAATCGTGACCGGAGT
*(m) IFNA4*	TATGTCCTCACAGCCAGCAG	TTCTGCAATGACCTCCATCA
*(m) IFNB1*	CCCAGTGCTGGAGAAATTGT	CCCTATGGAGATGACGGAGA
*(m) CXCL1*	TCTCCGTTCCTTGGGGACAC	CCACACTCAAGAATGGTCGC
*(m) CXCL2*	TCCAGGTCAGTTAGCCTTGC	CGGTCAAAAAGTTTGCCTTG
*(IAV) M*	AGATGAGTCTTCTAACCGAGGTCG	TGCAAAAACATCTTCAAGTCTCTG

qPCR: quantitative polymerase chain reaction; CXCL: C-X-C motif chemokine ligand; EIF2AK2: eukaryotic translation initiation factor 2 alpha kinase 2; HPRT: hypoxanthine-guanine phosphoribosyltransferase; IFN: interferon; ISG: IFN stimulated gene; M: matrix; MxA: myxovirus resistance 1; OAS: 2′-5′-oligoadenylate synthetase.

## References

[1] Cumulative Number of Confirmed Human Cases for Avian Influenza A (H5N1) Reported to WHO. http://www.who.int/influenza/human_animal_interface/H5N1_cumulative_table_archives/en/.

[2] Baz M., Abed Y., Simon P., Hamelin M.E., Boivin G. (2010). Effect of the neuraminidase mutation H274Y conferring resistance to oseltamivir on the replicative capacity and virulence of old and recent human influenza A (H1N1) viruses. J. Infect. Dis..

[3] De Jong M.D., Tran T.T., Truong H.K., Vo M.H., Smith G.J., Nguyen V.C., Bach V.C., Phan T.Q., Do Q.H., Guan Y. (2005). Oseltamivir resistance during treatment of influenza A (H5N1) infection. N. Engl. J. Med..

[4] Hurt A.C., Holien J.K., Parker M., Kelso A., Barr I.G. (2009). Zanamivir-resistant influenza viruses with a novel neuraminidase mutation. J. Virol..

[5] González-Navajas J.M., Lee J., David M., Raz E. (2012). Immunomodulatory functions of type I interferons. Nat. Rev. Immunol..

[6] Takeuchi O., Akira S. (2009). Innate immunity to virus infection. Immunol. Rev..

[7] Diamond M.S., Farzan M. (2013). The broad-spectrum antiviral functions of IFIT and IFITM proteins. Nat. Rev. Immunol..

[8] Wang B.X., Fish E.N. (2012). The yin and yang of viruses and interferons. Trends Immunol..

[9] Jia D., Rahbar R., Chan R.W., Lee S.M., Chan M.C., Wang B.X., Baker D.P., Sun B., Peiris J.S., Nicholls J.M. (2010). Influenza virus non-structural protein 1 (NS1) disrupts interferon signaling. PLoS ONE.

[10] Liedmann S., Hincius E.R., Anhlan D., McCullers J.A., Ludwig S., Ehhardt C. (2014). New virulence determinants contribute to the enhanced immune response and reduced virulence of an influenza A virus A/PR8/34 variant. J. Infect. Dis..

[11] Szretter K.J., Gangappa S., Belser J.A., Zeng H., Chen H., Matsuoka Y., Sambhara S., Swayne D.E., Tumpey T.M., Katz J.M. (2009). Early control of H5N1 influenza virus replication by the type I interferon response in mice. J. Virol..

[12] García-Sastre A. (2011). Induction and evasion of type I interferon responses by influenza viruses. Virus Res..

[13] Quinlivan M., Zamarin D., García-Sastre A., Cullinane A., Chambers T., Palese P. (2005). Attenuation of Equine Influenza Viruses though Truncations of the NS1 Protein. J. Virol..

[14] Salvatore M., Basler C.F., Parisien J.P., Horvath C.M., Bourmakina S., Zheng H., Muster T., Palese P., García-Sastre A. (2002). Effects of influenza A virus NS1 protein on protein expression: the NS1 protein enhances translation and is not required for shutoff of host protein synthesis. J. Virol..

[15] Bornholdt Z.A., Prasad B.V. (2008). X-ray structure of NS1 from a highly pathogenic H5N1 influenza virus. Nature.

[16] Bornholdt Z.A., Prasad B.V. (2006). X-ray structure of influenza virus NS1 effector domain. Nat. Struct. Mol. Biol..

[17] Liu J., Lynch P.A., Chien C.Y., Montelione G.T., Krug R.M., Berman H.M. (1997). Crystal structure of the unique RNA-binding domain of the influenza virus NS1 protein. Nat. Struct. Biol..

[18] Guo Z., Chen L.M., Zeng H., Gomez J.A., Plowden J., Fujita T., Katz J.M., Donis R.O., Sambhara S. (2007). NS1 protein of influenza A virus inhibits the function of intracytoplasmic pathogen sensor, RIG-I. Am. J. Respir. Cell. Mol. Biol..

[19] Mibayashi M., Martínez-Sobrido L., Loo Y.M., Cárdenas W.B., Gale M., García-Sastre A. (2007). Inhibition of retinoic acid-inducible gene I-mediated induction of beta interferon by the NS1 protein of influenza A virus. J. Virol..

[20] Li Y., Chen Z.Y., Wang W., Baker C.C., Krug R.M. (2001). The 3′-end-processing factor CPSF is required for the splicing of single-intron pre-mRNAs in vivo. RNA.

[21] Nemeroff M.E., Barabino S.M., Li Y., Keller W., Krug R.M. (1998). Influenza virus NS1 protein interacts with the cellular 30 kDa subunit of CPSF and inhibits 3′end formation of cellular pre-mRNAs. Mol. Cell..

[22] Haye K., Burmakina S., Moran T., García-Sastre A., Fernandez-Sesma A. (2009). The NS1 protein of a human influenza virus inhibits type I interferon production and the induction of antiviral responses in primary human dendritic and respiratory epithelial cells. J. Virol..

[23] Solórzano A., Webby R.J., Lager K.M., Janke B.H., García-Sastre A., Richt J.A. (2005). Mutations in the NS1 protein of swine influenza virus impair anti-interferon activity and confer attenuation in pigs. J. Virol..

[24] Wacheck V., Egorov A., Groiss F., Pfeiffer A., Fuereder T., Hoeflmayer D., Kundi M., Popow-Kraupp T., Redlberger-Fritz M., Mueller C.A. (2010). A novel type of influenza vaccine: Safety and immunogenicity of replication-deficient influenza virus created by deletion of the interferon antagonist NS1. J. Infect. Dis..

[25] Hale B.G., Batty I.H., Downes C.P., Randall R.E. (2008). Binding of influenza A virus NS1 protein to the inter-SH2 domain of p85 suggests a novel mechanism for phosphoinositide 3-kinase activation. J. Biol. Chem..

[26] Hale B.G., Jackson D., Chen Y.H., Lamb R.A., Randall R.E. (2006). Influenza A virus NS1 protein binds p85beta and activates phosphatidylinositol-3-kinase signaling. Proc. Natl. Acad. Sci. USA.

[27] Shin Y.K., Li Y., Liu Q., Anderson D.H., Babiuk L.A., Zhou Y. (2007). SH3 binding motif 1 in influenza A virus NS1 protein is essential for PI3K/Akt signaling pathway activation. J. Virol..

[28] Hincius E.R., Hennecke A.K., Gensler L., Nordhoff C., Anhlan D., Vogel P., McCullers J.A., Ludwig S., Ehhardt C. (2012). A single point mutation (Y89F) within the non-structural protein 1 of influenza A viruses limits epithelial cell tropism and virulence in mice. Am. J. Pathol..

[29] Gupta S., Yan H., Wong L.H., Ralph S., Krolewski J., Schindler C. (1996). The SH2 domains of Stat1 and Stat2 mediate multiple interactions in the transduction of IFN-alpha signals. EMBO J..

[30] Wallweber H.J.A., Tam C., Franke Y., Starovasnik M.A., Lupardus P.J. (2014). Structural basis of IFNα receptor recognition by TYK2. Nat. Struct. Mol. Biol..

[31] Hale B.G., Kerry P.S., Jackson D., Precious B.L., Gray A., Killip M.J., Randall R.E., Russell R.J. (2010). Structural insights into phosphoinositide 3-kinase activation by the influenza A virus NS1 protein. Proc. Natl. Acad. Sci. USA.

[32] Liu Q., Wang S., Ma G., Pu J., Forbes N.E., Brown E.G., Liu J.H. (2009). Improved and simplified recombineering approach for influenza virus reverse genetics. J. Mol. Genet. Med..

[33] Martínez-Sobrido L., García-Sastre A. (2010). Generation of recombinant influenza virus from plasmid DNA. J. Vis. Exp..

[34] Rogers E., Wang B.X., Cui Z., Rowley D.R., Ressler S.J., Vyakarnam A., Fish E.N. (2012). WFDC1/ps20: A host factor that influences the neutrophil response to murine hepatitis virus (MHV) 1 infection. Antiviral Res..

[35] Durbin J.E., Hackenmiller R., Simon M.C., Levy D.E. (1996). Targeted disruption of the mouse Stat1 gene results in compromised innate immunity to viral disease. Cell.

[36] García-Sastre A., Durbin R.K., Zheng H., Palese P., Gertner R., Levy D.E., Durbin J.E. (1998). The role of interferon in influenza virus tissue tropism. J Virol..

[37] Alvarado J.J., Tarafdar S., Yeh J.I., Smithgall T.E. (2014). Interaction with the Src homology (SH3-SH2) region of the Src-family kinase Hck structures the HIV-1 Nef dimer for kinase activation and effector recruitment. J. Biol. Chem..

[38] Matskova L.V., Helmstetter C., Ingham R.J., Gish G., Lindholm C.K., Ernberg I., Pawson T., Winberg G. (2007). The Shb signalling scaffold binds to and regulates constitutive signals from the Epstein-Barr virus LMP2A membrane protein. Oncogene.

[39] Mazumder E.D., Jardin C., Vogel B., Heck E., Scholz B., Lengenfelder D., Sticht H., Ensser A. (2012). A molecular model for the differential activation of STAT3 and STAT6 by the herpesviral oncoprotein tip. PLoS ONE.

[40] Devaux P., Priniski L., Cattaneo R. (2013). The measles virus phosphoprotein interacts with the linker domain of STAT1. Virology.

[41] Shaw M.L., Garcia-Sastre A., Palese P., Basler C.F. (2004). Nipah virus V and W proteins have a common STAT1-binding domain yet inhibit STAT1 activation from the cytoplasmic and nuclear compartments, respectively. J. Virol..

[42] Garcin D., Marq J.B., Strahle L., le Mercier P., Kolakofsky D. (2002). All four Sendai Virus C proteins bind Stat1, but only the larger forms also induce its mono-ubiquitination and degradation. Virology.

[43] Anjum S., Afzal M.S., Ahmad T., Aslam B., Waheed Y., Shafi T., Qadri I. (2013). Mutations in the STAT1-interacting domain of the hepatitis C virus core protein modulate the response to antiviral therapy. Mol. Med. Rep..

[44] Lin W., Kim S.S., Yeung E., Kamegaya Y., Blackard J.T., Kim K.A., Holtzman M.J., Chung R.T. (2006). Hepatitis C virus core protein blocks interferon signaling by interaction with the STAT1 SH2 domain. J. Virol..

[45] Zurney J., Howard K.E., Sherry B. (2007). Basal expression levels of IFNAR and Jak-STAT components are determinants of cell-type-specific differences in cardiac antiviral responses. J. Virol..

[46] Long J.X., Peng D.X., Liu Y.L., Wu Y.T., Liu X.F. (2008). Virulence of H5N1 avian influenza virus enhanced by a 15-nucleotide deletion in the viral nonstructural gene. Virus Genes.

[47] Forbes N.E., Ping J., Dankar S.K., Jia J.J., Selman M., Keleta L., Zhou Y., Brown E.G. (2012). Multifunctional adaptive NS1 mutations are selected upon human influenza virus evolution in the mouse. PLoS ONE.

[48] Dankar S.K., Wang S., Ping J., Forbes N.E., Keleta L., Li Y., Brown E.G. (2011). Influenza A virus NS1 gene mutations F103L and M106I increase replication and virulence. Virol. J..

[49] Li S., Min J.Y., Krug R.M., Sen G.C. (2006). Binding of the influenza A virus NS1 protein to PKR mediates the inhibition of its activation by either PACT or double-stranded RNA. Virology.

[50] Bergmann M., Garcia-Sastre A., Carnero E., Pehamberger H., Wolff K., Palese P., Muster T. (2000). Influenza virus NS1 protein counteracts PKR-mediated inhibition of replication. J. Virol..

[51] Silverman R.H. (2007). Viral encounters with 2′,5′-oligoadenylate synthetase and RNase L during the interferon antiviral response. J. Virol..

[52] Narasaraju T., Yang E., Samy R.P., Ng H.H., Poh W.P., Liew A.A., Phoon M.C., van Rooijen N., Chow V.T. (2011). Excessive neutrophils and neutrophil extracellular traps contribute to acute lung injury of influenza pneumonitis. Am. J. Pathol..

[53] Perrone L.A., Plowden J.K., García-Sastre A., Katz J.M., Tumpey T.M. (2008). H5N1 and 1918 pandemic influenza virus infection results in early and excessive infiltration of macrophages and neutrophils in the lungs of mice. PLoS Pathog..

[54] Stock A.T., Smith J.M., Carbone F.R. (2014). Type I IFN suppresses Cxcr2 driven neutrophil recruitment into the sensory ganglia during viral infection. J. Exp. Med..

[55] Seo S.U., Kwon H.J., Ko H.J., Byun Y.H., Seong B.L., Uematsu S., Akira S., Kweon M.N. (2011). Type I interferon signaling regulates Ly6C(hi) monocytes and neutrophils during acute viral pneumonia in mice. PLoS Pathog..

